# Temporally correlated fluctuations drive epileptiform dynamics

**DOI:** 10.1016/j.neuroimage.2016.11.034

**Published:** 2017-02-01

**Authors:** Maciej Jedynak, Antonio J. Pons, Jordi Garcia-Ojalvo, Marc Goodfellow

**Affiliations:** a Departament de Física i Enginyeria Nuclear, Universitat Politècnica de Catalunya, Terrassa, Spain; b Department of Experimental and Health Sciences, Universitat Pompeu Fabra, Parc de Recerca Biomèdica de Barcelona, Barcelona, Spain; c College of Engineering, Mathematics and Physical Sciences, University of Exeter, Exeter, UK; d Centre for Biomedical Modelling and Analysis, University of Exeter, Exeter, UK; e EPSRC Centre for Predictive Modelling in Healthcare, University of Exeter, Exeter, UK

**Keywords:** Epilepsy, Ictogenesis, Neural mass models, Jansen-Rit model, Nonlinear dynamics, Stochastic effects, Ornstein‐Uhlenbeck noise

## Abstract

Macroscopic models of brain networks typically incorporate assumptions regarding the characteristics of afferent noise, which is used to represent input from distal brain regions or ongoing fluctuations in non-modelled parts of the brain. Such inputs are often modelled by Gaussian white noise which has a flat power spectrum. In contrast, macroscopic fluctuations in the brain typically follow a $1/f^{b}$ spectrum. It is therefore important to understand the effect on brain dynamics of deviations from the assumption of white noise. In particular, we wish to understand the role that noise might play in eliciting aberrant rhythms in the epileptic brain.

To address this question we study the response of a neural mass model to driving by stochastic, temporally correlated input. We characterise the model in terms of whether it generates “healthy” or “epileptiform” dynamics and observe which of these dynamics predominate under different choices of temporal correlation and amplitude of an Ornstein-Uhlenbeck process. We find that certain temporal correlations are prone to eliciting epileptiform dynamics, and that these correlations produce noise with maximal power in the *δ* and *θ* bands. Crucially, these are rhythms that are found to be enhanced prior to seizures in humans and animal models of epilepsy. In order to understand why these rhythms can generate epileptiform dynamics, we analyse the response of the model to sinusoidal driving and explain how the bifurcation structure of the model gives rise to these findings. Our results provide insight into how ongoing fluctuations in brain dynamics can facilitate the onset and propagation of epileptiform rhythms in brain networks. Furthermore, we highlight the need to combine large-scale models with noise of a variety of different types in order to understand brain (dys-)function.

## Highlights

• We use Jansen-Rit model under stochastic and periodic driving to study ictogenesis.
• Correlation time of the driving noise modulates characteristic of the model's output.
• Driving with delta/theta rhythms is most prone to initiate epileptic activity.

## 1 Introduction

Epilepsy is a prevalent neurological disorder characterised by the recurrence of spontaneous seizures. Seizures predominantly arise amidst a backdrop of otherwise healthy brain activity and are often accompanied by salient changes in electrographic activity as measured, for example, on the electroencephalogram (EEG). There is much we do not understand about why seizures occur, and contributing factors exist across multiple temporal and spatial scales (Lytton, 2008, Wendling et al., 2015). Here we focus upon a large spatial scale of interconnected brain regions since this is the scale at which clinical signs and symptoms emerge, and clinical data are most often recorded. At this scale, deficits can be observed both in the dynamics of brain regions (Valentín et al., 2005, Iannotti et al., 2016) and the connections between brain regions (O'Muircheartaigh et al., 2012). Thus recent focus has been placed on the role that large-scale brain networks play in epilepsy (Spencer, 2002, Kramer and Cash, 2012, Richardson, 2012, van Diessen et al., 2013). A fundamental, unanswered question in this context is how seizures emerge and spread in such networks (Goodfellow et al., 2011, Terry et al., 2012, Petkov et al., 2014, Goodfellow, 2016, Aksenova et al., 2007, Villa and Tetko, 2010).

Understanding seizures as emergent dynamics in brain networks is a challenging endeavour. However, mathematical models of brain dynamics can be used to study the mechanisms underlying the generation of seizures (Suffczynski et al., 2006, Lytton, 2008, Wendling et al., 2015). Previous work has focused on the types of dynamics that could underpin transitions from healthy EEG to seizure EEG, such as changes in model parameters (bifurcations), co-existence of healthy and abnormal states (bistability) or more complex spatiotemporal dynamics (Wendling et al., 2002, Lopes da Silva et al., 2003, Breakspear et al., 2006, Goodfellow et al., 2011, Rothkegel and Lehnertz, 2011, Baier et al., 2012, Goodfellow and Glendinning, 2013). The bifurcation route into seizures relies on a (relatively) slow time scale change in the brain that drives it into an alternate (pathological) state, whereas the bistability paradigm relies on a (fast) perturbation-induced transition from the healthy to pathological state. However, any of these scenarios can be assumed to occur amidst a backdrop of ongoing brain dynamics, which could additionally influence transitions into seizures.

Modelling studies of seizure onset typically lump the “background” dynamics of the brain into stochastic fluctuations. These fluctuations have most often been assumed to have a flat power spectrum (i.e. Gaussian white noise) (Lopes da Silva et al., 1974, Pons et al., 2010, Victor et al., 2011, Roberts and Robinson, 2012, Touboul et al., 2011, Petkov et al., 2014, Garnier et al., 2015), which can be motivated by the assumption that ongoing activity of the brain is so complex that no single frequency dominates. However, analysis of spectra of brain signals (for example scalp EEG) reveals ongoing brain dynamics to be characterised by a $1/f^{b}$ relationship (Buzsáki and Draguhn, 2004), with prominent frequencies appearing concomitantly with different brain states (Niedermeyer and LopesdaSilva, 2005, Buzsáki and Draguhn, 2004, Freeman et al., 2000). In the epileptic brain, abnormal (“epileptiform”) rhythms such as spikes or slow waves can also be present, even during interictal periods (Valentín et al., 2014, Karoly et al., 2016). In particular, in humans an increase of power in the delta band has been observed in MEG (Gupta et al., 2011) and EEG (Sadleir et al., 2011) recordings preceding absence seizures and pathological slow rhythms can be observed in interictal or preictal periods associated with focal epilepsies (Valentín et al., 2014, Tao et al., 2011, Lee et al., 2000). In animal models of epilepsy, electrophysiological recordings performed in the preictal phase have revealed an increase of power in the delta (Sitnikova and van Luijtelaar, 2009), and delta and theta (Van Luijtelaar et al., 2011) bands.

We therefore need to better understand the response of neuronal populations to afferent rhythms and stochastic fluctuations with a variety of dynamics, including those that can be approximated by noise yielding a realistic $1/f^{b}$ power spectrum, and those that contain dominant rhythms observed in the epileptic brain. A natural choice for the generation of such noise is the Ornstein-Uhlenbeck (OU) process, which exhibits a Lorentzian power spectrum. The spectral distribution in the OU process can be tuned through temporal correlations (i.e. “colour”) of the resulting noise, therefore modelling alternative spectral compositions. OU noise has also been associated with the integration of background synaptic activity acting upon a neuron (Destexhe and Rudolph, 2004). Recent studies of OU processes driving neural models have investigated the effects of coloured noise on temporal distributions of neuronal spiking (Braun et al., 2015, da Silva and Vilela, 2015) and the generation of multimodal patterns of alpha activity (Freyer et al., 2011). In addition, networks of spiking neurons (Sancristóbal et al., 2013) and of neuronal populations (Jedynak et al., 2015) have been shown to generate realistic $1/f^{b}-\mathrm{like}$ spectra when driven by OU noise, or more complex dynamics when subjected to driving at specific frequencies (Spiegler et al., 2011, Malagarriga et al., 2015). However, we lack an understanding of the ways in which non-white noise or rhythmic perturbations interact with neuronal populations to produce epileptiform dynamics.

Here, we study the effect of temporally correlated noise and rhythmic driving on the generation of epileptiform dynamics. Our starting point is a neural mass model that represents canonical interactions between populations of neurons in a region of brain tissue. Such models have been shown to be capable of generating pathological spiking dynamics reminiscent of seizure activity (Jansen et al., 1993, Jansen and Rit, 1995, Wendling et al., 2000, Grimbert and Faugeras, 2006). We classify the dynamics of this model by assessing variations of the signal around its time-averaged value, thus distinguishing between “healthy” and epileptiform dynamics. We then study the response of the system to prototypical coloured noise (an OU process) and identify an interval of temporal correlations for which noise can more readily elicit epileptiform dynamics. We show that this region is bounded on the one hand by noise intensity being insufficient to generate spikes, and on the other by bursting and transitions to an alternative rhythmic state, previously used to model healthy dynamics (the alpha rhythm). Analysing the spectrum of noise in this interval reveals it to contain high power in low (2–8 Hz) frequencies. In order to understand why such frequencies can drive epileptiform rhythms, we study periodic perturbations in a deterministic version of the model. Our analysis shows that driving the deterministic model using frequencies in this band causes epileptiform dynamics to predominate. We show how consideration of the bifurcation structure of the model can shed light on these observations, which in turn highlight the need to consider a fuller analysis of the repertoire of dynamics in the model beyond the genesis of epileptiform rhythms. Our findings elucidate potential mechanisms by which healthy or epileptiform rhythms present in certain regions of the brain can cause the onset of aberrant dynamics in connected regions.

## 2 Materials and methods

### 2.1 Jansen and Rit model

In order to study the dynamics of regions of brain tissue, we use a neural mass model of a canonical circuit of interacting neuronal populations (Jansen et al., 1993, Jansen and Rit, 1995). The populations considered are pyramidal neurons, excitatory interneurons and inhibitory interneurons. The dynamics of these populations is governed by a linear transformation that converts presynaptic spiking activity to changes in postsynaptic membrane potential (PSP) and a nonlinear transformation of net membrane potential to an efferent firing rate.

The linear transformation is given by the following convolution:

(1) $$y(t)=\int_{0}^{\infty }h(t\prime )s_{\mathrm{in}}(t-t\prime )dt\prime ,$$

where $s_{\mathrm{in}}(t)$ is the spike rate of activity afferent to the population, *y*(*t*) gives the dynamics of the PSP, and *h*(*t*) describes the way in which membrane potentials respond to an activating impulse. *h*(*t*) equals zero for t<0 and otherwise is given for excitatory and inhibitory connections with the following equations:

(2) $$h_{e}(t)={\mathrm{Aate}}^{-\mathrm{at}},$$

(3) $$h_{i}(t)={\mathrm{Bbte}}^{-\mathrm{bt}},$$

where *A* and *B* are the maximum excitatory and inhibitory PSPs, respectively, and *a* and *b* are time constants of these responses. They follow from lumped contributions of all dilatory effects that include synaptic kinetics, dendritic signal propagation and leak currents (Wilson and Cowan, 1972, Freeman, 1972, Amari, 1974, Nunez, 1974, Lopes da Silva et al., 1974).

Eq. (1) can be rewritten, using Eq. (2), as a second order ordinary differential equation (ODE):

(4) $$\frac{d^{2}y(t)}{{\mathrm{dt}}^{2}}+2a\frac{\mathrm{dy}(t)}{\mathrm{dt}}+a^{2}y(t)=\mathrm{Aa}\cdot s_{\mathrm{in}}(t),$$

Similarly, by using Eq. (3) one can find a corresponding representation for inhibitory population dynamics.

Conversion of net membrane potential to efferent spiking is given by the following sigmoid function:

(5) $$s_{\mathrm{out}}(y)=\mathrm{Sigm}(y)=\frac{2e_{0}}{1+e^{r(\nu_{0}-y)}},$$

where $s_{\mathrm{out}}(y)$ is a firing rate of a spike train outgoing from the population, *y* is its momentary total PSP (in general, time dependent), 2e_0_ is the maximum firing rate, *ν*_0_ is the PSP for which half maximum of the firing rate is reached, and *r* determines steepness (and thus nonlinearity) of this transformation.

**Fig. 1:**
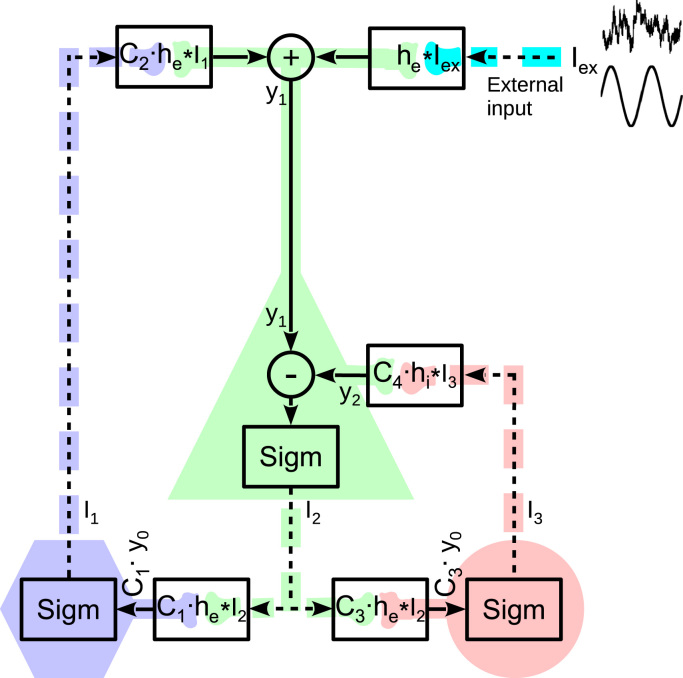
A scheme of the Jansen-Rit model of a cortical column that comprises three neuronal populations. A population of pyramidal neurons is marked with green, and populations of excitatory and inhibitory interneurons with blue and red, respectively. Somata are depicted with the triangle, hexagon and circle. Continuous lines stand for dendritic processing and dashed ones for axonal processing. A dot means multiplication and a star operator denotes convolution. Cyan indicates lumped external input from sub-cortical and cortico-cortical structures. The black circuit depicts an analytic description of the underlying structure of a cortical column. See text for details.

The two described transformations allow to model circuits of interconnected neuronal populations. A circuit corresponding to a Jansen-Rit model of a cortical column is shown in black in Fig. 1.

The equations above lead to a full description of circuit dynamics (Fig. 1) as follows:

$$\{\begin{array}{cc} {\overset{¨}{y}}_{0}(t)+2a{\overset{˙}{y}}_{0}(t)+a^{2}y_{0}(t)=\mathrm{Aa}\;\mathrm{Sigm}[y_{1}(t)-y_{2}(t)] & \;(6)\\ {\overset{¨}{y}}_{1}(t)+2a{\overset{˙}{y}}_{1}(t)+a^{2}y_{1}(t)=\mathrm{Aa}\{I_{\mathrm{ex}}(t)+C_{2}\;\mathrm{Sigm}[C_{1}y_{0}(t)]\} & \;(7)\\ {\overset{¨}{y}}_{2}(t)+2b{\overset{˙}{y}}_{2}(t)+b^{2}y_{2}(t)=\mathrm{Bb}\{C_{4}\;\mathrm{Sigm}[C_{3}y_{0}(t)]\} & \;(8) \end{array}$$

where *y*_0_ is proportional to excitatory PSPs induced on both populations of interneurons, *y*_1_ is a net excitatory PSP induced on the population of pyramidal neurons and *y*_2_ is an inhibitory PSP on this population. Subsequently $y_{1}-y_{2}$ is the resultant PSP on this population, which following previous studies is assumed to be proportional to the measured EEG. We set parameters of the neural mass model to typically used values as given in Jansen and Rit (1995): $e_{0}=2.5\;s^{-1}$, $v_{0}=6\;\mathrm{mV}$, $r=0.56\;{\mathrm{mV}}^{-1}$, A=3.25mV, B=22mV, $a=100\;s^{-1}$, $b=50\;s^{-1}$, $C_{1}=135$, $C_{2}=108$, $C_{3}=C_{4}=33.75$.

### 2.2 Driving of the model

**Fig. 2:**
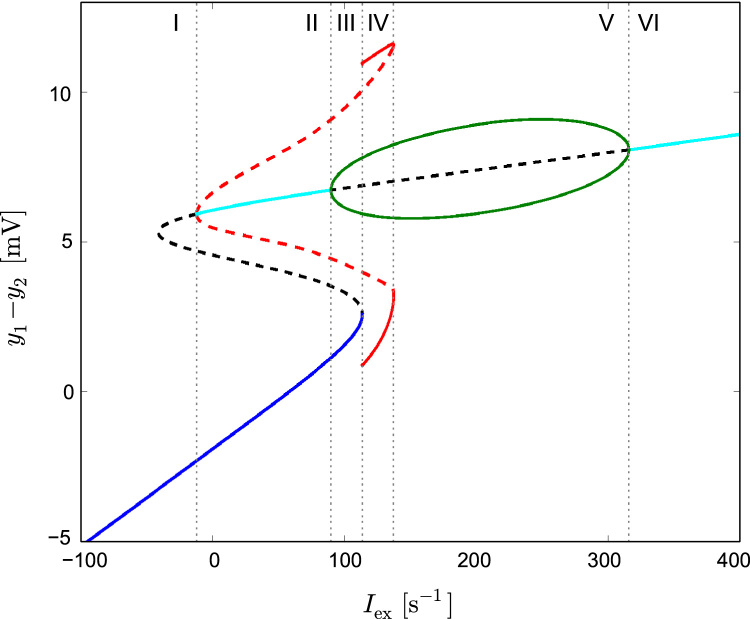
Bifurcation diagram of the Jansen-Rit model defined in Eqs. (6)–(8). Parameters of the model were set to biologically plausible values proposed in Jansen and Rit (1995). The X axis shows external, constant input to the pyramidal population $I_{\mathrm{ex}}=p$. The Y axis shows net postsynaptic potential on this population: $y_{1}-y_{2}$. Continuous (dashed) lines represent stable (unstable) solutions. Cyan and blue denote a node and a focus, respectively, and green and red indicate alpha and epileptiform limit cycles, respectively. Vertical, grey dotted lines divide the diagram to six regimes (denoted by roman numerals) of qualitatively distinct dynamical properties. See text for details.

$I_{\mathrm{ex}}(t)$ in Eq. (7) represents external input to the microcircuit, lumping together cortico-cortical and sub-cortical afferents. The effect of $I_{\mathrm{ex}}(t)$ on the dynamics of the model is the focus of our study. Previous studies have sought to understand the dynamics of the model by examining the effect of $I_{\mathrm{ex}}$ as a bifurcation parameter and found certain values of this parameter to lead to epileptiform spiking (Jansen and Rit, 1995, Grimbert and Faugeras, 2006, Spiegler et al., 2011, Touboul et al., 2011). In Fig. 2 we recreate with XPPAUT (Ermentrout, 2002) the results of Grimbert and Faugeras (2006), illustrating the invariant sets of the model that exist for different, time invariant values of $I_{\mathrm{ex}}$. To ease subsequent interpretations of dynamics invoked by different choices of temporally varying $I_{\mathrm{ex}}(t)$, we briefly review the different dynamic regimes that are possible in this model. Although in Fig. 2 we plot a range of $I_{\mathrm{ex}}$ that includes negative values (region I in Fig. 2), we focus on positive values of $I_{\mathrm{ex}}$, since only these are biologically plausible. The regime marked II in Fig. 2 spans for $-12.15\;s^{-1}<p<89.83\;s^{-1}$. It is a bistable regime that contains two stable fixed points: a node (blue) and a focus (cyan). At $p=89.83\;s^{-1}$ the focus transitions to a limit cycle (green) in a supercritical Hopf bifurcation. This limit cycle has its characteristic frequency close to 10 Hz, and has therefore previously been used to model the alpha rhythm of the brain (henceforth referred to as “alpha limit cycle”). The regime marked III is also bistable, however here the two stable solutions are the node (blue) and the alpha limit cycle (green). At $p=113.58\;s^{-1}$ the stable node ceases to exist in a saddle-node on invariant circle (SNIC) bifurcation that creates a limit cycle reminiscent of epileptiform spikes, henceforth referred to as “epileptiform limit cycle” (continuous red line, Fig. 2). The frequency of this limit cycle ranges from 0 Hz at its creation to ∼5 Hz at its termination point. Region IV in Fig. 2 denotes a bistable regime in which the epileptiform limit cycle coexists with the alpha limit cycle. Regime V starts at $p=137.38\;s^{-1}$, where the epileptiform limit cycle vanishes in a fold of limit cycles. In regime V the alpha limit cycle is the only stable solution. It ceases to exist in a supercritical Hopf bifurcation at $p=315.70\;s^{-1}$, where the last regime, marked with VI, starts. The focus (cyan) remains the only stable solution there.

Here we focus on the dynamics of the microcircuit under the influence of noisy or rhythmic perturbations from other regions of the brain. We therefore decompose $I_{\mathrm{ex}}(t)$ into a time invariant part *p* and a zero-mean, time dependent component *u*(*t*) as follows:

(9) $$I_{\mathrm{ex}}(t)=p+u(t)$$

*p* determines the average working point of the system in the landscape of dynamical regimes as shown in Fig. 2. Following from our previous study (Jedynak et al., 2015) we choose a default value of $p=89s^{-1}$, placing the system close to the Hopf bifurcation. *u*(*t*) represents deterministic or stochastic perturbations: for the former, we use $u(t)=\overset{˜}{A}\sin(\frac{2\pi }{T}t+\phi )$, for the latter, we use an Ornstein-Uhlenbeck (OU) process. This noise, $\xi_{\mathrm{ou}}$, is derived from the solution of the following linear stochastic differential equation:

(10) $$\frac{d\xi_{\mathrm{ou}}}{\mathrm{dt}}=-\frac{\xi_{\mathrm{ou}}}{\tau }+\frac{\sqrt{2D}}{\tau }\xi_{w}(t)$$

where $\xi_{w}(t)$ is a random variable representing Gaussian white noise with zero mean and correlation $\langle \xi_{w}(t)\xi_{w}(t\prime )\rangle =\delta (t-t\prime )$ and *τ* is correlation time of the OU noise. The standard deviation of this noise in the steady state is:

(11) $$\sigma_{\mathrm{ou}}=\sqrt{\frac{D}{\tau }}$$

The variables $I_{\mathrm{ex}}$, *p*, *u*, $\xi_{\mathrm{ou}}(t)$ and $\sigma_{\mathrm{ou}}$ represent firing rates of spike trains and therefore are expressed in $s^{-1}$. The intensity of the noise can be defined as the product of its stationary variance (accounting for amplitudes of random fluctuations) and its correlation time (accounting for persistence of the fluctuations) (Carlo Laing, 2010). In the notation adopted here, the intensity defined in this way is given by *D*. Finally, the power spectrum of the OU noise is given by the Lorentzian function:

(12) $$S_{\mathrm{ou}}(f)=\frac{2D}{1+4\pi^{2}\tau^{2}f^{2}}$$

In order to study how the frequency content of OU noise relates to traditionally defined EEG frequency bands (i.e. δ,θ,α,β,γ) we quantify the fraction of total spectral power of the noise (characterised with correlation time *τ*) contained in a certain frequency window, bounded by $f_{\min},f_{\max}$:

(13) $$E\left(\tau ,f_{\min},f_{\max}\right)=\frac{2}{P_{\mathrm{tot}}}\cdot \int_{f_{\min}}^{f_{\max}}S_{\mathrm{ou}}(f)\mathrm{df}={\frac{2}{\pi }\arctan(2\pi \tau f)|}_{f_{\min}}^{f_{\max}}$$

where the normalisation factor $P_{\mathrm{tot}}$ yields the total power and equals $\int_{-\infty }^{\infty }S_{\mathrm{OU}}(f)\mathrm{df}=\frac{D}{\tau }$. The factor 2 in front of the integral follows from taking into account power transmitted in both the positive and negative frequency bands.

### 2.3 Classification of model dynamics

As previously described (Jansen and Rit, 1995, Grimbert and Faugeras, 2006, Spiegler et al., 2010, Touboul et al., 2011), the model can display “healthy” or “epileptiform” rhythms depending upon its parametrisation and the nature of its input, $I_{\mathrm{ex}}$ (see Eq. (9)). By considering the bifurcation diagram shown in Fig. 2, we define epileptiform dynamics as those corresponding to the epileptiform limit cycle, and healthy dynamics as any of the other regimes. The latter comprises either noise-driven fluctuations around the node, or oscillations with frequency close to 10 Hz (alpha oscillations) due to the presence of, or proximity to, the limit cycle generated by the Hopf bifurcation.

**Fig. 3:**
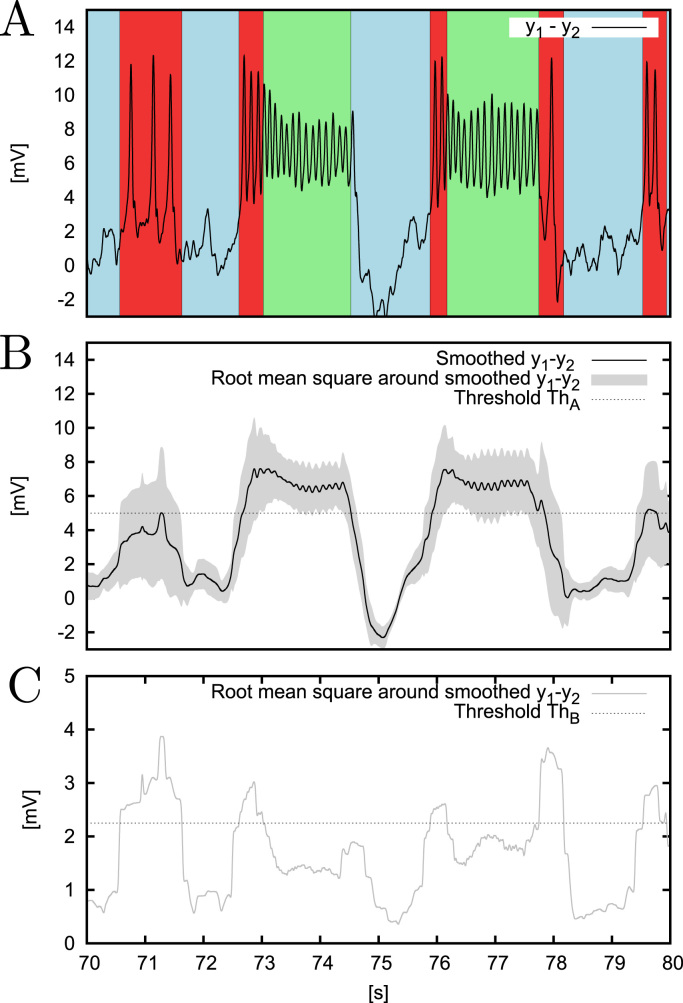
Methodology for classification of dynamics. Panel A shows $y_{1}-y_{2}$ obtained from 10 s of stochastic simulation for $p=89\;s^{-1}$, $\tau =10^{-0.5}\;s$, $\sigma_{\mathrm{ou}}=50\;s^{-1}$. Background colours indicate the type of activity assigned with the classification algorithm. Red stands for epileptiform dynamics, green for alpha oscillations, and blue for random fluctuations around the node. Panel B shows a smoothed version of the $y_{1}-y_{2}$ signal from panel A, obtained with a running mean computed within a 0.4-second-long sliding window. The dashed line denotes the ${\mathrm{Th}}_{A}=5\;\mathrm{mV}$ threshold, which is used to discriminate between stochastic fluctuations around the node (smoothed $y_{1}-y_{2}\leq {\mathrm{Th}}_{A}$) and alpha oscillations (smoothed $y_{1}-y_{2}>{\mathrm{Th}}_{A}$). Grey marks root mean square of $y_{1}-y_{2}$ around its smoothed version (${\mathrm{RMS}}_{\left(y_{1}-y_{2}\right)}$). This value is shown in panel C in grey along with the ${\mathrm{Th}}_{B}=2.25\;\mathrm{mV}$ threshold, which is used to identify epileptiform dynamics (when ${\mathrm{RMS}}_{\left(y_{1}-y_{2}\right)}>{\mathrm{Th}}_{B}$).

Our classification of the output of the Jansen-Rit cortical column in these three categories is depicted in Fig. 3. The classification is established via the following algorithm: first, a moving average of the model's output $y_{1}-y_{2}$ is computed with a sliding window of length 0.4 s. This window is long enough to sufficiently smooth out the signal (see Fig. 3B) and thus allow for estimation of its variability (details below), and short enough to mark transitions between dynamical regimes with good temporal accuracy (see Fig. 3A).

Second, the root mean square (${\mathrm{RMS}}_{\left(y_{1}-y_{2}\right)}$) of the $y_{1}-y_{2}$ signal around this mean is obtained. When this quantity is high, variations of the signal are rapid and/or have a high-amplitude, which are features of the epileptiform limit cycle. Therefore, we set a threshold ${\mathrm{Th}}_{B}=2.25\;\mathrm{mV}$ (dashed line in Fig. 3C) that establishes the value of ${\mathrm{RMS}}_{\left(y_{1}-y_{2}\right)}$ above which a specific time point of the signal is classified as being in epileptiform dynamics. Otherwise, we compare the smoothed $y_{1}-y_{2}$ signal with the threshold value ${\mathrm{Th}}_{A}=5\;\mathrm{mV}$ (dashed line in Fig. 3B), which separates the focus from the node along the $y_{1}-y_{2}$ axis (c.f. Fig. 2). If the smoothed signal is greater than ${\mathrm{Th}}_{A}$, we classify a data point as alpha oscillations, if it is smaller, we classify it as noisy fluctuations around the node. Note that this methodology is valid also for deterministic conditions, as in Section 3.3. The thresholds ${\mathrm{Th}}_{A}$ and ${\mathrm{Th}}_{B}$ as well as the window length have been set such that resulting classification complies with inspection by eye. The attractor-based classification method described above is adequate in our case, since our model attractors can be sufficiently distinguished by amplitude. For other types of models, or for the analysis of experimental data, adding frequency information to aid the classification might be beneficial, although purely temporal classifications have been found to be sufficient in some cases (Kramer et al., 2012).

### 2.4 Computational simulation

We integrated the system using the stochastic Heun scheme (Toral and Colet, 2014) with a time step equal to 10^−4^ s, and we stored every tenth point of the simulation. For each value of noise correlation time *τ* and stationary standard deviation $\sigma_{\mathrm{ou}}$ we performed 10 simulations, each with different realisation of the noise and we averaged the results. Each simulation was 111 s long. The first 10 s were discarded and one second buffered the sliding window. In the deterministic system, the model was simulated for 111 s, with 100 s of transient discarded and one second buffering the sliding window. This means that the effective time courses used in the deterministic analysis were 10 s long, which corresponds to the longest period of the driving sinusoid that we utilised.

## 3 Results

### 3.1 Noise induced epileptiform dynamics

**Fig. 4:**
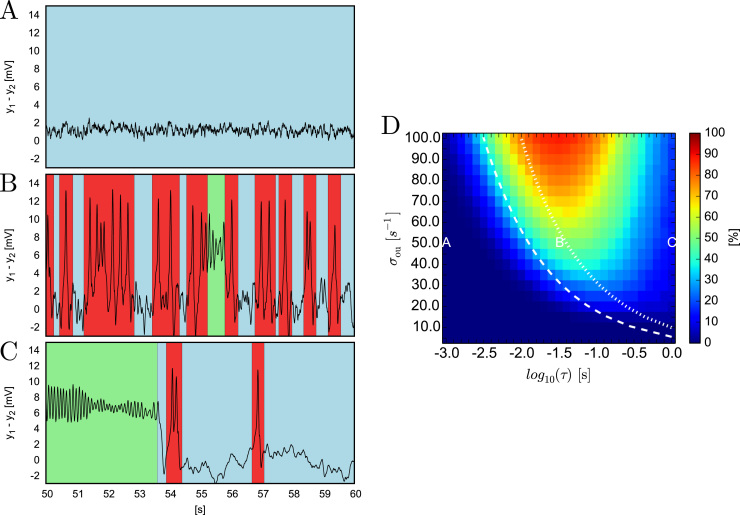
Response of the Jansen-Rit model to driving with the Ornstein-Uhlenbeck (OU) noise. The left panels of the figure show example outputs (time courses of $y_{1}-y_{2}$) produced by the model under driving with the OU noise characterised with correlation time *τ* equal to 10^−3^ s (panel A), $10^{-1.5}\;s$ (panel B) and 10^0^ s (panel C). Background colours mark periods of random fluctuations around the node (blue), epileptiform dynamics (red) and alpha activity (green). In all these cases stationary standard deviation of the noise $\sigma_{\mathrm{ou}}$ was equal to $50\;s^{-1}$ and *p* was set to $89\;s^{-1}$. Panel D shows the fraction of time that the system spent in epileptiform dynamics as a function of the noise correlation time *τ* (varied along the X axis in logarithmic scale) and the noise stationary standard deviation $\sigma_{\mathrm{ou}}$ (varied along the Y axis). Locations of the red letters A,B and C mark settings in which time traces shown in panels A,B and C were obtained. The white lines denote points of equal values of noise intensity *D*: the dashed line marks $D=\sqrt{1000}\;s^{-1}$ and the dotted one marks $D=100\;s^{-1}$. In all cases initial conditions corresponded exactly to the node.

Simulations of the model under different values of the correlation time, *τ*, of the driving Ornstein-Uhlenbeck (OU) noise reveal qualitatively different dynamics (Fig. 4). For weakly correlated noise (low values of *τ*) stochastic fluctuations around the node predominate (Fig. 4A). For intermediate temporal correlations epileptiform rhythms are more often observed (Fig. 4B), whilst at larger correlation times the model displays mainly node and alpha activities (Fig. 4C). These results suggest that epileptiform dynamics are more readily observed for noise with intermediate correlation times. In order to systematically study this effect, simulations of the model were performed for different values of *τ* and standard deviation of the noise, $\sigma_{\mathrm{ou}}$. For each simulation, we measured the fraction of the total time that the system spent in epileptiform dynamics (Fig. 4D).

Fig. 4D shows that for large enough values of the standard deviation $\sigma_{\mathrm{ou}}$ epileptiform dynamics arise for an intermediate value of the noise correlation time. As $\sigma_{\mathrm{ou}}$ decreases, the interval of values of *τ* for which epileptiform dynamics predominates is shifted to larger values. The intensity of OU noise, *D*, as described in Eq. (11) is overlaid in white dashed lines on Fig. 4D. It can be seen that the onset of epileptiform dynamics for intermediate values of *τ* coincides with constant values of *D*. This means that in order to generate epileptiform dynamics, the noise generated by the OU process should have sufficient intensity, regardless of its power and correlation time. However, this simple relationship does not hold for $\tau ≳10^{-1.5}\;s$. The system more often displays alpha oscillations for large correlation times ($\tau ≳10^{-0.5}\;s$) than for small correlation times. Supplementary Fig. S1 illustrates the fractions of time that the system spends in alpha oscillations and in the node attractor. In order to test the generalisability of these results, we performed equivalent simulations under alternative choices of parameters *a* and *b*, such that the presence of the attractor representing epileptiform dynamics was preserved. We found that although changes in the bifurcation diagram occurred (Fig. S2 in Supplementary Materials), the value of *τ* maximising the presence of epileptiform dynamics remained the same (Fig. S3 in Supplementary Materials). Increasing the value of *σ* still further can be seen to increase the range of *τ* over which epileptiform dynamics are elicited (Fig. S4 in Supplementary Materials).

### 3.2 Relationship to brain rhythms

**Fig. 5:**
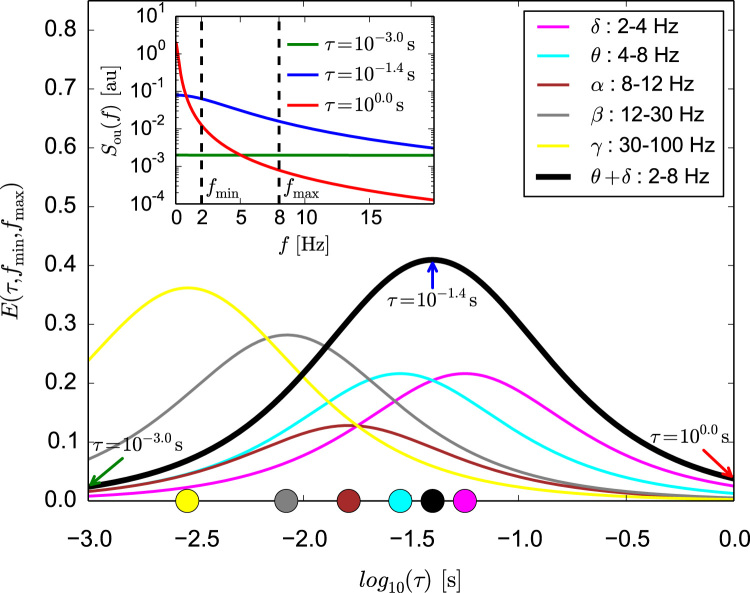
Distribution of spectral power in frequency bands of standard brain rhythms and dependence of location of maximum power on noise correlation time *τ*. Evaluation of the $E(\tau ,f_{\min},f_{\max})$ function (see Eq. (13)) of an Ornstein-Uhlenbeck noise characterised with correlation time *τ* within a frequency range $f_{\min},f_{\max}$ is plotted for fixed frequency ranges that correspond to distinct brain rhythms: *δ* (2–4 Hz, magenta), *θ* (4–8 Hz, cyan), *α* (8–12 Hz, brown), *β* (12–30 Hz, grey), *γ* (30–100 Hz, yellow) and combined *δ*+*θ* (2–8 Hz, black). Units on the Y axis express fraction of the spectral power of the noise characterised with *τ* contained within the $f_{\min},f_{\max}$ range. Correlation time of the noise *τ* varies along the X axis. The inset illustrates the meaning of $E(\tau ,f_{\min},f_{\max})$. It shows an example theoretical power spectrum of the Ornstein-Uhlenbeck noise calculated for $\tau =10^{-3.0}\;s$ (green), $\tau =10^{-1.4}\;s$ (blue) and $\tau =10^{0}\;s$ (red). In each case stationary variance $\frac{D}{\tau }$ was set to an arbitrary value 1 s^−2^. Dashed vertical lines mark the $f_{\min}=2\;\mathrm{Hz}$, $f_{\max}=8\;\mathrm{Hz}$ range, for which the black plot shown in the main panel was derived from Eq. (13). Green, blue and red arrows on the main plot indicate values of the $E(\tau ,f_{\min},f_{\max})$ function that correspond to these spectra. The value indicated by the blue arrow is highest (in this case it corresponds to the maximum), which follows from the fact that the area below the blue curve, limited by $f_{\min}$ and $f_{\max}$ in the inset is greater that area set by either red, or green curves. Coloured circles on the X axis indicate values of *τ* corresponding to maxima of $E(\tau ,f_{\min},f_{\max})$: $\tau =10^{-2.54}\;s$ for *γ*, $\tau =10^{-2.08}\;s$ for *β*, $\tau =10^{-1.79}\;s$ for *α*, $\tau =10^{-1.55}\;s$ for *θ*, $\tau =10^{-1.25}\;s$ for *δ*, and $\tau =10^{-1.4}\;s$ for *δ* + *θ*.

To relate these findings to underlying frequency components of brain rhythms we studied how OU processes with different correlation times distribute their power in different frequencies. In order to do this we used Eq. (13) to quantify the fraction of power deposited by the noise (characterised with correlation time, *τ*) in a given frequency window ($f_{\min},f_{\max}$). Evaluation of this function for $f_{\min}$ and $f_{\max}$ set according to boundaries of traditionally defined EEG frequency bands (δ,θ,α,β,γ) is shown in Fig. 5. For each frequency band, the location of the maximum of the E function (Eq. (13)) represents the value of noise correlation time *τ* that maximises spectral power of the noise within that band. Values of *τ* corresponding to these maxima are indicated with coloured circles on the X axis of Fig. 5. They demonstrate that the choice of noise correlation time $\tau =10^{-1.55}\;s$ maximises spectral power in the *θ* band (cyan). Furthermore, $\tau =10^{-1.25}$ maximises spectral power in the *δ* band (magenta). Experimental studies suggest that enhancement of rhythms falling to these two bands may precede occurrence of epileptiform activity (Gupta et al., 2011, Sadleir et al., 2011, Van Luijtelaar et al., 2011, Sitnikova and van Luijtelaar, 2009). We therefore combine *δ* and *θ* bands together and find that spectral power within this *δ*+*θ* band is maximised for $\tau =10^{-1.4}$. As shown in the previous section (Fig. 4), this value of *τ* coincides with correlation times of the driving noise for which epileptic spiking is most prevalent. Therefore, we speculate that rhythms around the *θ* band (4–8 Hz) or in the wider δ+θ band (2–8 Hz) are particularly prone to eliciting epileptiform dynamics in the model.

### 3.3 Periodic driving in the deterministic system

In order to test this prediction, we analysed the response of the system to harmonic driving $u(t)=\overset{˜}{A}\sin(\frac{2\pi }{T}t+\phi )$. We systematically varied the amplitude $\overset{˜}{A}$, period *T* and phase *ϕ* of the harmonic driving, and quantified the dynamics of the model. It has previously been shown that the Jansen-Rit model displays a variety of dynamics, caused by rhythmic driving, including periodicity, quasi-periodicity and chaos (Malagarriga et al., 2015, Spiegler et al., 2011). In this case, however, we narrow our interest to whether the activity resembles epileptiform dynamics, alpha oscillations, or fluctuations around the node, and therefore apply the same classification algorithm as in the stochastic system (see Methods and Fig. 3). We focus on elucidating values of amplitude and frequency for which healthy or epileptiform dynamics are observed.

**Fig. 6:**
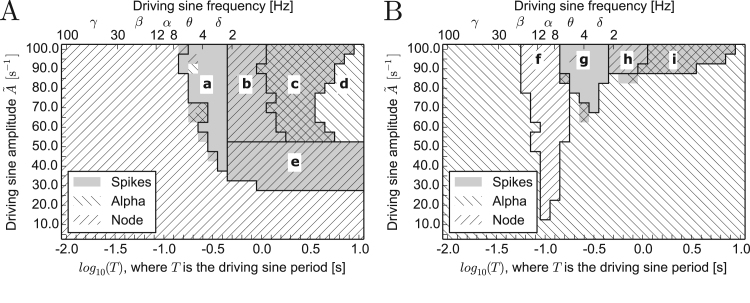
Phase diagram showing the different dynamical regimes resulting from oscillatory driving with varying amplitude and period. The response of the Jansen-Rit model under harmonic driving was classified as either a node (oblique stripes from top-right to bottom-left), alpha activity (oblique stripes from top-left to bottom-right), or epileptiform dynamics (grey). This classification was conducted for varying driving amplitude $\overset{˜}{A}$, displayed on Y axes, and driving period *T*, displayed on X axes in logarithmic (bottom) and linear (top) scales. Ranges and names of typical brain rhythms are denoted on the linear scale. In general, different dynamical regimes might coexist, therefore patterns overlap. Panel A corresponds to initial conditions set exactly to the node and panel B to initial conditions set exactly to alpha oscillations. In both cases $p=89\;s^{-1}$. Black lines divide the diagram into distinct regimes, annotated with letters.

Fig. 6 shows the presence of each of these dynamics when the amplitude and period of the driving harmonic signal are varied. Alpha oscillations and the node solution are encoded with oblique stripes (top-right to bottom-left for the node and top-left to bottom-right for alpha) and epileptiform dynamics are encoded with grey. Fig. 6A corresponds to settings where initial conditions were set exactly to the node, whereas Fig. 6B corresponds to initial conditions exactly at the focus. Fig. 6A demonstrates that for fast periodic driving ($T≲10^{-0.8}\;s$), the initial node dynamics are preserved and epileptiform rhythms are not elicited even when the driving amplitude is large. On the other hand, for very slow driving ($T>10^{0.5}\;s$) and sufficiently high amplitude ($\overset{˜}{A}>50\,\;s^{-1}$), alpha oscillations dominate (regime “d”). Similarly to the stochastic case, epileptiform dynamics prevail for intermediate periods of the driving and sufficiently large amplitude (regime “a”). An exemplary time course corresponding to this case is provided in supplementary Fig. S5. For initial conditions set to alpha oscillations, Fig. 6B demonstrates that neither fast ($T≲10^{-1.2}\;s$) nor slow driving, characterised with an amplitude not exceeding a limit value, causes transitions away from the initial dynamics. Similarly to the node initial conditions, intermediate values of *τ* give rise to epileptiform dynamics (regime “g”). In particular, exclusively epileptiform dynamics occur when driving frequencies correspond to either *δ* or *θ* rhythms.

Driving with frequencies of ∼10 Hz leads to a resonance effect, causing an escape from alpha oscillations to the node. This effect is present in regime “f” and the corresponding time course is shown in Fig. S6, in Supplementary Materials. This resonance results in long-term node dynamics. However, when excitability of the model is increased (an increase in parameter *p*), this escape from the alpha attractor is followed by epileptic activity (see Supplementary Fig. S7). Figs. 6A and B were obtained for *ϕ*=0. We note that alternative choices of *ϕ* did not alter the results of Fig. 6A. However, we did identify an effect of altering phase in that the resonance regime (“f” in Fig. 6B) is slightly narrower when the driving sinusoid is shifted by the phase $\phi =+\frac{\pi }{2}$. In these conditions no resonance appears for $T=10^{-1.2}\;s$, and for $T=10^{-1.1}\;s$ and $\overset{˜}{A}\in [70\;s^{-1},\;85\;s^{-1}]$. For other phase shifts this effect of resonance attenuation is not prominent, or does not occur, but the lower boundaries of regimes “g”, “h” and “i” can be extended towards smaller values of $\overset{˜}{A}$ for some non-zero phase shifts.

These effects can be understood from the structure of the bifurcation diagram of the model (Grimbert and Faugeras, 2006) shown in Fig. 2. In particular, transient periods of intensive spiking (bursting), interleaved with periods of quiescence are observed when a slowly varying input periodically crosses the bifurcation and leads the system to alternate between regimes III and IV. In this case, the system switches between the node (denoted by blue in regime III in Fig. 2) and epileptiform spikes (continuous red in regime IV). These dynamics are represented in Fig. 6 as combined spiking+node activity in regimes “b” and “h”. In this case, although the driving amplitudes can be high enough to enter regime V, alpha oscillations are not observed, because driving is too fast and the system does not have time to converge to these oscillations. Furthermore, regime “e” in Fig. 6A marks driving that is slow enough and characterised by amplitudes high enough to cross the excitability threshold (enter regime IV in Fig. 2) - thus eliciting bursts of spikes - but at the same time not large enough to enter regime V of alpha oscillations. An exemplary bursting time course, corresponding to this regime is provided in Supplementary Materials, in Fig. S8.

Slow driving with sufficiently high amplitude moves the system through all dynamic regimes and overshoots the epileptiform spiking regime to regime V where alpha oscillations are the only existing dynamics. In this case, the system displays the effects of hysteresis. For the upswing of the driving sinusoid all three dynamical regimes are displayed: from the node in regimes II and III (blue in Fig. 2), through epileptiform dynamics in regime IV (continuous red in Fig. 2), to alpha oscillations in regime V (green in Fig. 2). During the downswing phase of the driving, however, the system remains in quasistatic conditions in the alpha attractor, so in the bistable regimes IV and III it exhibits alpha oscillations (green in Fig. 2) and in the bistable regime II it remains on the focus (cyan in Fig. 2). This hysteresis loop is closed when driving with a sufficiently high amplitude moves the system to, or sufficiently close, regime I, where the system relaxes to the node (blue in Fig. 2). This effect occurs in regimes “c” and “i” combining all three types of dynamics. An exemplary time course corresponding to regime “c” is provided in Supplementary Materials, in Fig. S9. For smaller driving frequencies the system remains in alpha oscillations and does not revert to the node (regime “d”). A similar effect is observed for initial conditions set to the focus (panel Fig. 6B). These effects explain why stochastic driving with power concentrated in low frequencies promotes alpha oscillations of the system (as described in Section 3.1). Supplementary Materials, Fig. S10 shows how slow driving, characterised with a sufficiently high amplitude, pushes the system deeper into the alpha limit cycle, thereby increasing the amplitude of alpha oscillations. We note that these model regimes are also physiologically relevant, since slow (0.25 Hz) driving has been shown to lead to an increased power in the *α* band (Bayer et al., 2011, Jedynak et al., 2015) and bursting following a slow quasi-harmonic pattern may occur in the early ictal phase of seizures (Alarcon et al., 1995).

## 4 Discussion

In this study we investigated the effect of rhythmic driving and coloured noise on the generation of epileptiform dynamics in a neural mass model. We found that epileptiform dynamics are more readily elicited by noise with certain temporal correlations. By exploring the composition of OU noise in different frequency bands and driving of the model with sinusoidal rhythms, we discovered that simulated epileptiform discharges are more easily generated by rhythms in the delta and theta frequency bands. Thus we suggest that the local microcircuit interactions embodied by the model can give rise to emergent dynamics that leave it prone to generating epileptiform rhythms when bombarded by afferent spiking with particular rhythmic properties.

Experimental and clinical findings lend support to this hypothesis. Interictal focal slow activity in the delta or delta-theta bands has been shown to be present in a majority of invasive recordings from people with temporal lobe epilepsy (Valentín et al., 2014), and it lateralises with regions of seizure onset (Valentín et al., 2014), in particular in neocortical temporal lobe epilepsy (Tao et al., 2011). Thus slow rhythms are associated with epileptic brain networks (Tao et al., 2011). Our modelling results lead us to hypothesise that such rhythms could also be the cause of onset of seizures in such networks. Indeed, slow rhythms are also observed in invasive recordings at seizure onset in focal epilepsies (Lee et al., 2000, Jiménez-Jiménez et al., 2015). Slow rhythms have also been observed in association with generalised epilepsies in both clinical and experimental data. Sitnikova and van Luijtelaar (2009) observed an increase of delta activity prior to onset of spike-wave-discharges in the WAG/Rij rodent model and Van Luijtelaar et al. (2011) reported an increase of delta and theta rhythms in the preictal phase of brain activity in the same animal model. The frequency of the alpha rhythm has also been shown to be lower in people with epilepsy compared to control subjects (Larsson and Kostov, 2005). Our results suggest a potential mechanism of propagation of abnormal dynamics in large-scale brain networks: a local network generating abnormal rhythms could induce the propagation of this activity in connected brain regions. Future extensions to our work could examine explicitly the dynamics of networks of neural masses in order to investigate conditions for propagation or restriction of epileptiform activity.

The epileptic brain is increasingly being thought of and studied in terms of networks (Berg et al., 2010, Goodfellow et al., 2011, Richardson, 2012, Petkov et al., 2014, Sanz-Leon et al., 2015, Naze et al., 2015). Understanding seizure generation in networks is a difficult task since seizures represent emergent transitions in dynamics due to both the underlying connectivity structure of the network and the intrinsic dynamics of individual nodes (Terry et al., 2012, Goodfellow, 2016). To simplify this situation, in our study we separated intrinsic node and network effects, considering the effects of temporally structured afferent activity to a node. Our observations that certain rhythms preferably generate epileptiform dynamics arise from an interplay between the presence of different invariant sets (Fig. 2) and the time scale of fluctuations in $I_{\mathrm{ex}}$. For example, on the node branch, close to the epileptiform limit cycle, slow variations in afferents can allow the epileptiform limit-cycle to appear and, if the amplitude of these fluctuations is in a certain interval, the system can also converge to this attractor, therefore displaying epileptiform rhythms. By uncovering these phenomena in the deterministic system, we are able to better understand the ways in which stochastic fluctuations with power in certain frequencies could cause transitions in dynamics and ultimately lead to epileptiform activity.

In our study we used a set of parameters for the Jansen-Rit model that give rise to dynamics relevant to the study of healthy brain function such as the alpha rhythm as well as pathological dynamics (Jansen et al., 1993, Jansen and Rit, 1995, Wendling et al., 2000). Previous studies have used bifurcation analyses to demonstrate how the arrangement of invariant sets varies in parameter space (Spiegler et al., 2010, Touboul et al., 2011), and have studied the response of neural mass models to driving by rhythmic pulses (Spiegler et al., 2011) and white noise (Wendling et al., 2000). Our work advances on these previous studies by quantifying the effect that temporally correlated noise and rhythmic input have in terms of the generation of epileptiform spiking, which led us to hypothesise a role for low-frequency brain rhythms in the generation of seizures. We therefore demonstrated the importance of non-white noise in the context of bifurcations of neural mass models to uncover the mechanisms underlying brain (dys-)function. The chosen parameter set enabled us to study the effect that different afferent dynamics have on the generation of these dynamics, and we further demonstrated that variations in the arrangement of attractors did not affect the optimal time scale for induction of epileptiform dynamics.

Our study utilised a neural mass model that is capable of generating epileptiform dynamics via a SNIC bifurcation, which has been shown to be a generic onset mechanism for a variety of epileptiform rhythms, including spike-wave discharges and focal seizures (Marten et al., 2009, Jirsa et al., 2014). We therefore believe our results to be applicable in the context of both focal and generalised epilepsies. In future work it will be important to study the effects of coloured noise in a variety of different models, such as extensions to the neural mass model (Wendling et al., 2002, Goodfellow et al., 2011) that can generate alternative dynamics, networks of neural masses, or networks of canonical models (Lopes da Silva et al., 2003, Benjamin et al., 2012, Goodfellow and Glendinning, 2013, Jirsa et al., 2014). It will be interesting to ascertain, for example, the conditions for propagation or restriction of epileptiform activity or whether certain epilepsies with specific emergent dynamics are susceptible to specific afferent rhythms for the generation of seizures.

## 5 Conclusion

The mechanisms underpinning the generation of seizures are imperfectly understood. In this work we have shown that the temporal correlation of signals afferent to neuronal populations may play a critical role in the initiation of epileptiform dynamics. The reasons for this can be understood in terms of the dynamical properties of these populations, in particular from the arrangement in parameter space of a variety of dynamical regimes. We therefore highlight the necessity of moving beyond white noise driving in computational studies of epilepsy.

## Appendix A Supplementary data

Application 1

## Footnotes

Appendix A Supplementary data associated with this article can be found in the online version at doi:10.1016/j.neuroimage.2016.11.034.
