# Supplementary material for: Temporally correlated fluctuations drive epileptiform dynamics
Source: Neuroimage. 2017 Feb 1;146:188–96. doi: 10.1016/j.neuroimage.2016.11.034 (PMC5353705; doi:10.1016/j.neuroimage.2016.11.034)
Supplement: Application 1 [file mmc1.pdf]

Supplementary Materials: Temporally correlated fluctuations drive epileptiform dynamics

Maciej Jedynak, Antonio J. Pons, Jordi Garcia-Ojalvo, Marc Goodfellow

Fig. S1 complements Fig. 4 from the main text. It corresponds to initial conditions set exactly to the node and it shows relative total times that the system spends in each of its attractors: the node (panel A), epileptic-like spiking (panel B) and alpha oscillations (panel C). For clarity panel Fig.4D from the main text has been replotted here (panel Fig. S1B). Fig. S1 corresponds to settings discussed in the main text, that is noise intensities $\sigma_{\rm ou} \in [5\ {\rm s}^{-1}, 100\ {\rm s}^{-1}]$, and $a = 100\ {\rm s}^{-1}, b = 50\ {\rm s}^{-1}$.

Fig. S2 complements the bifurcation diagram showed in Fig. 2 in the main text. It shows bifurcation diagrams obtained for varied values of parameters a and b. Increasing b to 52.5 $\rm s^{-1}$ leads to disappearance of the spiking attractor, therefore we did not study that case here. Decreasing b to 45 $\rm s^{-1}$ (panel A) leads to the spiking limit cycle existing for a range of $I_{\rm ex} = p$ values wider than in the case presented in the main text (where $b = 50\ {\rm s}^{-1}$). Moreover, for the most part this limit cycle does not coexist with any other stable solution. A similar effect is observed for decreased $a = 95\ {\rm s}^{-1}$ (panel B), wherein the regime of the spiking limit cycle exists for a range of $I_{\rm ex} = p$ values only slightly wider than for a and b settings used in the main text (where $a = 100\ {\rm s}^{-1}$). Also, this limit cycle begins at a lower $I_{\rm ex} = p$ value (101.06 $\rm s^{-1}$). Furthermore, similarly to the conditions of decreased b, here bistability is heavily reduced. The last case corresponds to increased $a = 110\ {\rm s}^{-1}$ (panel C). Here, the regime of spiking oscillations is reduced and the alpha limit cycle does not exist at all, although bistability is maintained (between the spiking limit cycle and the focus).

Fig. S3 shows how the changes in bifurcation structure mentioned above affect the relative total time that the system spends in epileptic-like spiking. Values of parameters a and b corresponding to panels A-C are set identically as in the case of Fig. S2. Panels A and B demonstrate that reducing bistability in the system (see again Fig. S2A,B) allows spikes to occur abundantly for high τ values. This is a result of the reduction of alpha activity in that regime. Spiking is more prevalent in Fig. S2B since in that case the spiking limit cycle is born at lower $I_{\rm ex} = p$ than in cases presented in the main text and in Fig. S3A. In the last case, due to the reduced width of the spiking attractor (see again Fig. S2C) epileptic-like activity is generally diminished in the system (panel C). Bistability is preserved here and due to noise the lack of the alpha limit cycle does not prevent the system from oscillating around the focus. This leads to reduction of spiking activity for high τ values, similarly as in the case presented in the main text. Fig. S4, similarly to Fig. S1 shows the relative total times that the system spends in each attractor, but it is concerned with higher $\sigma_{\rm ou}$ values than presented in the main text.

Figs. S5-S10 complement Fig. 6 from the main text. They show deterministic driving (panels A) and the corresponding response of the system (panels B) for typical cases discussed in section 3.3 in the main text. Red stripes in panels A correspond to $I_{\rm ex}$ values falling to the regime of coexistence of limit cycles. Fig. S5 shows the response of the system to driving with frequency falling to the δ range, that is ~ 3 Hz. In this case we observe phase locking between driving and the response, which results in continuous spiking. Figs. S6,S7 were obtained without the transient in order to demonstrate the resonance effect. To show it clearly, we turned on the sinusoidal driving only after one second of the simulation. Fig. S6 shows the escape from the focus due to driving coinciding with the natural frequency of the attractor (~ 10 Hz). After this escape the system remains in the basin of the node. It is not the case, however, when the system is rendered more excitable, for example by an increase of the constant component of external driving p to 113 $\rm s^{-1}$. In this case after escaping the alpha attractor the system may enter it transiently again, as well as displaying epileptiform dynamics. This effect is demonstrated in Fig. S7. Bursting, shown in Fig. S8B is characterised by periods of spiking interleaved with periods of quiescence, which occur for both the upswing and downswing of the

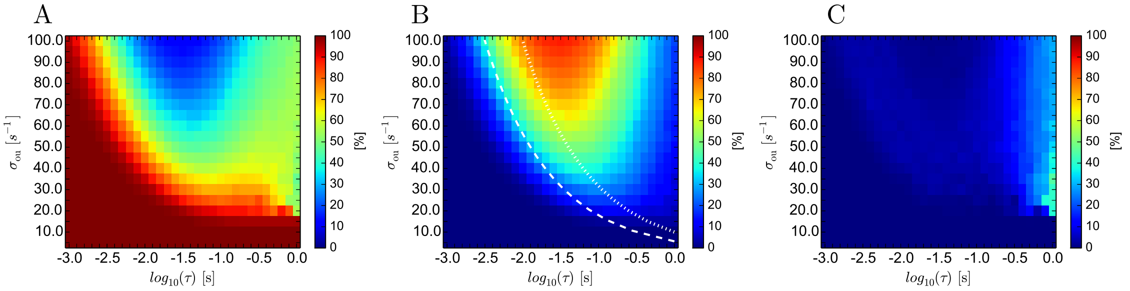

Figure S1: Fraction of time spent by the system in each of its attractors: fixed point (panel A), epileptic-like spiking activity (panel B) and alpha oscillations (panel C) shown as a function of autocorrelation time of the driving Ornstein-Uhlenbeck noise τ (varied along X axes) and stationary standard deviation of the noise σ_{ou} (varied along Y axes). The white lines in panel B denote points of equal values of noise intensity D: the dashed line marks $D=\sqrt{1000}$ s^{-1} and the dotted one marks $D = 100$ s^{-1}. The initial conditions corresponded exactly to the node.

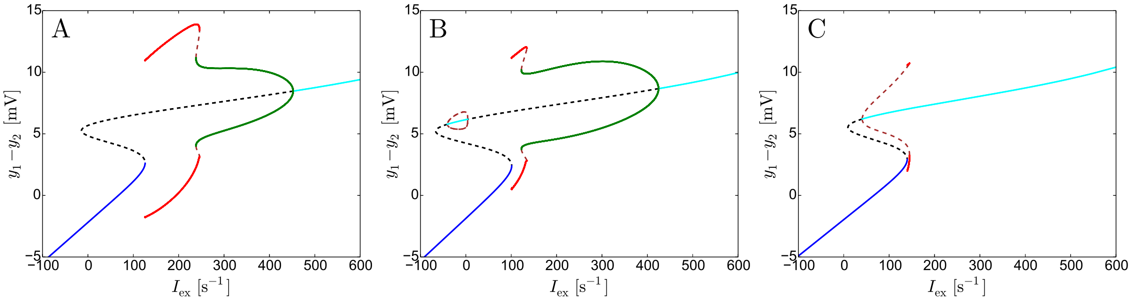

Figure S2: Bifurcation diagrams of the Jansen-Rit model obtained for varied values of parameters a and b: $a = 100$ $\mathrm{s}^{-1}, b = 45$ s^{-1} (panel A), $a = 95$ $\mathrm{s}^{-1}, b = 50$ s^{-1} (panel B), and $a = 110$ $\mathrm{s}^{-1}, b = 50$ s^{-1} (panel C). The X axes show external, constant input to the pyramidal population $I_{\mathrm{ex}} = p$. The Y axis shows net postsynaptic potential on this population: $y_1 - y_2$. Continuous (dashed) lines represent stable (unstable) solutions. Cyan and blue denote a node and a focus, respectively, and green and red indicate stable alpha and epileptiform limit cycles, respectively. Brown dashed line denotes unstable limit cycles.

driving sinusoid. Increasing the amplitude further from this case allows the system to enter a regime where alpha oscillations are the only stable solution. As a result during the downswing of the sinusoid the system remains in the alpha attractor. This effect of hysteresis is shown in Fig. S9. Further slowing down of the driving and a slight increase of its amplitude lead to the quasistatic dynamics (see Fig. S10), when the alpha attractor is never escaped from. This effect links to the stochastic case, when alpha oscillations occur mostly for high values of τ.

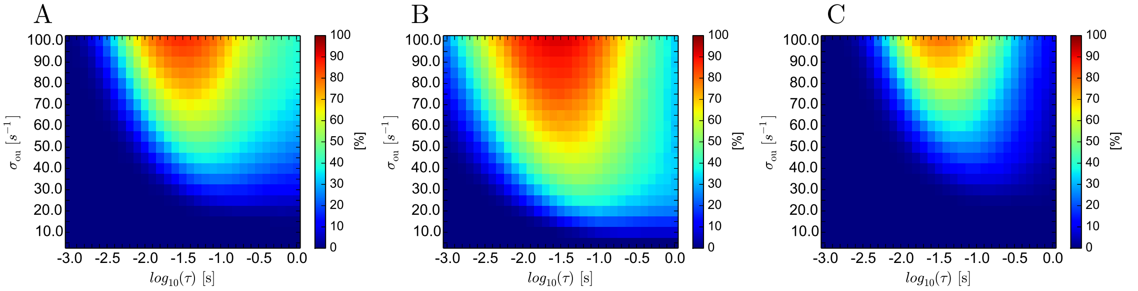

Figure S3: Fraction of time spent by the system on epileptic-like spiking, for varied values of parameters a and b: $a = 100$ s^{-1}, $b = 45$ s^{-1} (panel A), $a = 95$ s^{-1}, $b = 50$ s^{-1} (panel B), and $a = 110$ s^{-1}, $b = 50$ s^{-1} (panel C). Correlation time of the driving Ornstein-Uhlenbeck noise τ varies along X axes and stationary standard deviation of the noise $\sigma_{\rm ou}$ varies along Y axes.

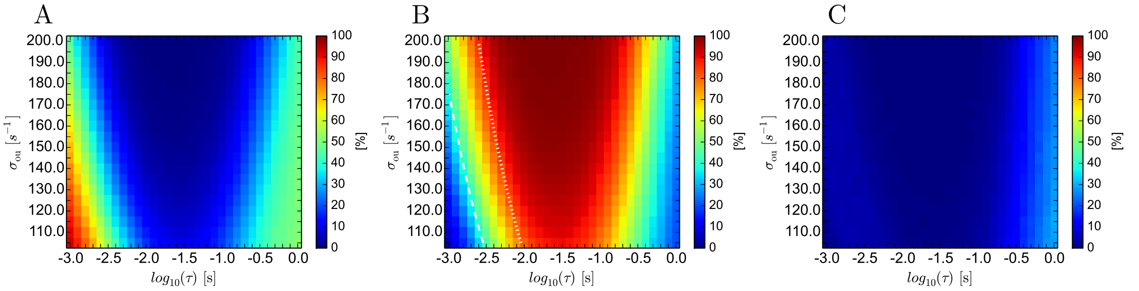

Figure S4: Fraction of time spent by the system in each of its attractors: fixed point (panel A), epileptic-like spiking activity (panel B) and alpha oscillations (panel C) shown as a function of autocorrelation time of the driving Ornstein-Uhlenbeck noise τ (varied along X axes) and stationary standard deviation of the noise $\sigma_{\rm ou}$ (varied along Y axes). The white lines in panel B denote points of equal values of noise intensity D: the dashed line marks $D=\sqrt{1000}$ s^{-1} and the dotted one marks $D = 100$ s^{-1}. In all cases initial conditions corresponded exactly to the focus.

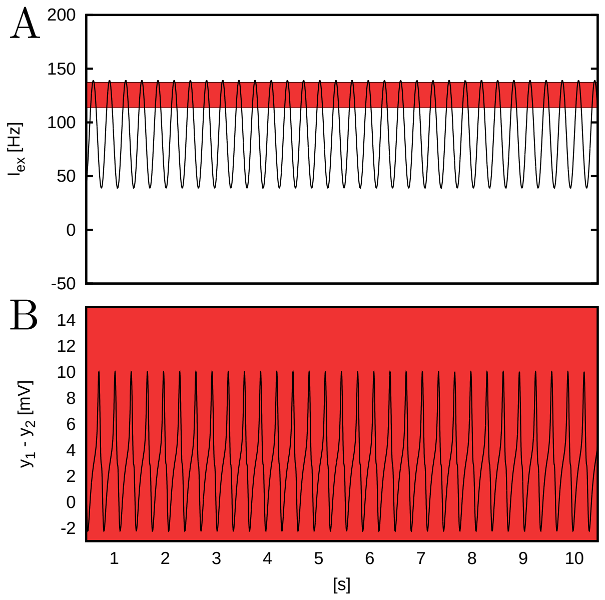

Figure S5: Pure spiking. The harmonic driving (shown in panel A) is characterised by the period $T = 10^{0.5}$ s and amplitude $\tilde{A} = 50$ s^{-1}. Constant component of the driving, p equals 89 s^{-1}. The red stripe in panel A marks input values corresponding to existence of the spiking limit cycle. Response of the system is shown in panel B. Initial conditions corresponded exactly to the node.

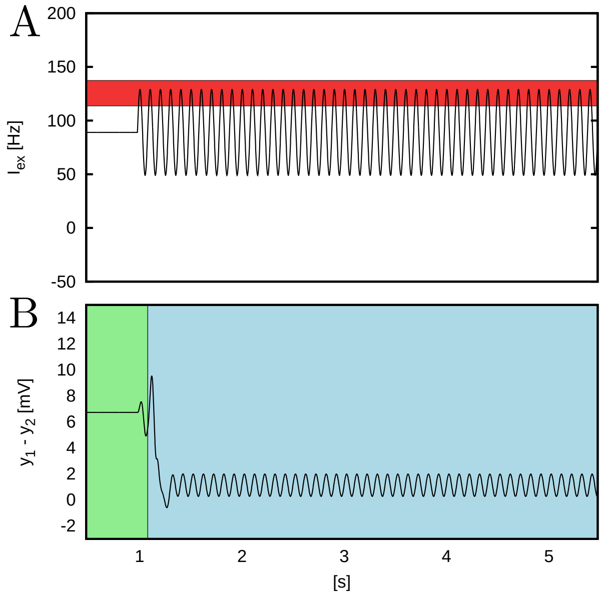

Figure S6: Alpha resonance. The harmonic driving (shown in panel A) is characterised by the period $T = 10^{-1}$ s and amplitude $\tilde{A} = 40$ s^{-1}. Constant component of the driving, p equals 89 s^{-1}. The red stripe in panel A marks input values corresponding to existence of the spiking limit cycle. Response of the system is shown in panel B. Initial conditions corresponded exactly to the focus.

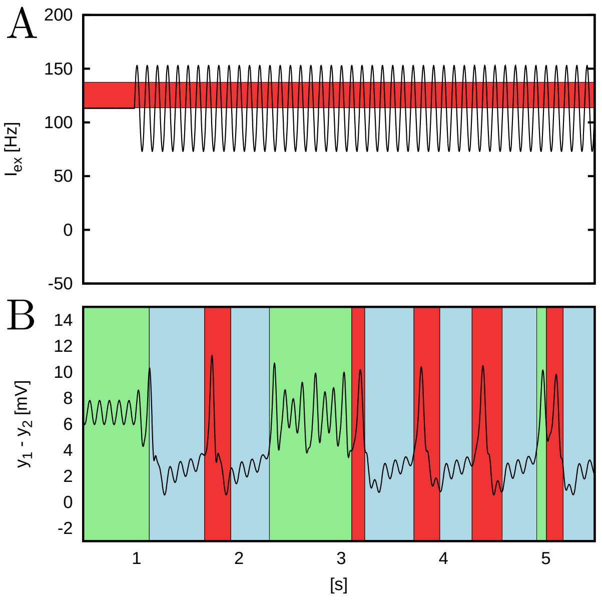

Figure S7: Alpha resonance in a highly excitable system. The harmonic driving (shown in panel A) is characterised by the period $T = 10^{-1}$ s and amplitude $\tilde{A} = 40$ s^{-1}. Constant component of the driving, p equals 113 s^{-1}. The red stripe in panel A marks input values corresponding to existence of the spiking limit cycle. Response of the system is shown in panel B. Initial conditions corresponded exactly to the focus.

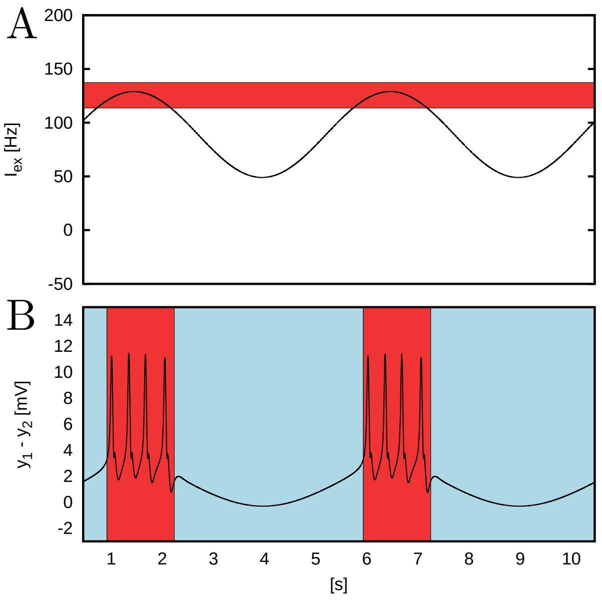

Figure S8: Bursting. The harmonic driving (shown in panel A) is characterised by the period $T = 10^{0.7}$ s and amplitude $\tilde{A} = 40$ s^{-1}. Constant component of the driving, p equals 89 s^{-1}. The red stripe in panel A marks input values corresponding to existence of the spiking limit cycle. Response of the system is shown in panel B. Initial conditions corresponded exactly to the node.

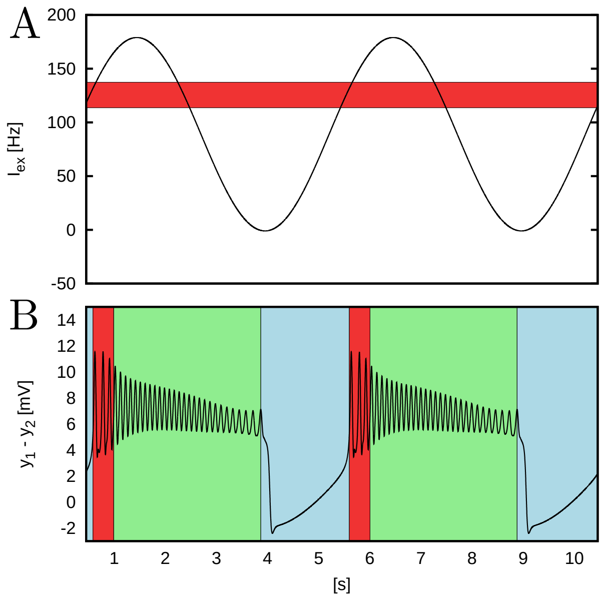

Figure S9: Hysteresis. The harmonic driving (shown in panel A) is characterised by the period $T = 10^{0.7}$ s and amplitude $\tilde{A} = 90$ s^{-1}. Constant component of the driving, p equals 89 s^{-1}. The red stripe in panel A marks input values corresponding to existence of the spiking limit cycle. Response of the system is shown in panel B. Initial conditions corresponded exactly to the node.

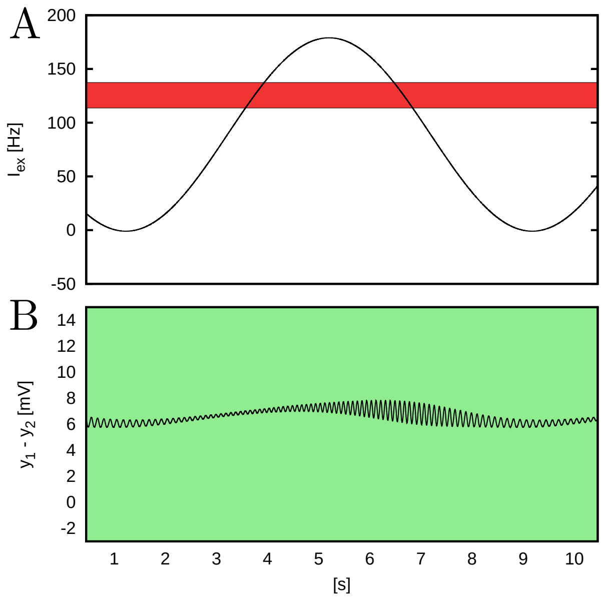

Figure S10: Quasistatic dynamics. The harmonic driving (shown in panel A) is characterised by the period $T = 10^{0.9}$ s and amplitude $\tilde{A} = 90$ s^{-1}. Constant component of the driving, p equals 89 s^{-1}. The red stripe in panel A marks input values corresponding to existence of the spiking limit cycle. Response of the system is shown in panel B. Initial conditions corresponded exactly to the node.
